# Community engagement processes in low- and middle-income countries health research settings: a systematic review of the literature

**DOI:** 10.1186/s12913-023-09466-9

**Published:** 2023-05-08

**Authors:** Zinhle Mthembu, John J. O. Mogaka, Moses J. Chimbari

**Affiliations:** 1 University of KwaZulu-Natal. College of Health Science, School of Nursing and Public Health, Howard College, 269 Mazisi Kunene Road, Berea, Durban 4041 South Africa; 2 Faculty of Humanities and Social Sciences, Anthropology and Development Studies, University of Zululand, 1 Main Road, Vulindlela, KwaDlangezwa 3886 South Africa; 3 Great Zimbabwe University, Masvingo, P.O. Box 1234, Zimbabwe

**Keywords:** Community engagement, Participation, Empowerment, Research settings, Low-and middle-income countries

## Abstract

**Background:**

Community Engagement is an important ethical imperative in research. Although substantial research emphasizes its real value and strategic importance, much of the available literature focuses primarily on the success of community participation, with little emphasis given to specific community engagement processes, mechanisms and strategies in relation to intended outcomes in research environments. The systematic literature review’s objective was to explore the nature of community engagement processes, strategies and approaches in health research settings in low- and middle-income countries.

**Methods:**

The systematic literature review design was informed by the Preferred Reporting Items for Systematic Reviews and Meta-Analyses guidelines. We searched for peer-reviewed, English-language literature published between January 2011 and December 2021 through three databases on the internet (PubMed, Web of Science and Google Scholar). The terms "community engagement," "community involvement," "participation," "research settings," and "low- and middle-income countries" were merged in the search.

**Results:**

The majority of publications [8/10] were led by authors from low- and middle-income countries, with many of them, [9/10] failing to continuously include important aspects of study quality. Even though consultation and information sessions were less participatory, articles were most likely to describe community engagement in these types of events. The articles covered a wide range of health issues, but the majority were concerned with infectious diseases such as malaria, human immunodeficiency virus, and tuberculosis, followed by studies on the environment and broader health factors. Articles were largely under-theorized.

**Conclusions:**

Despite the lack of theoretical underpinnings for various community engagement processes, strategies and approaches, community engagement in research settings was variable. Future studies should go deeper into community engagement theory, acknowledge the power dynamics underpin community engagement, and be more practical about the extent to which communities may participate.

**Supplementary Information:**

The online version contains supplementary material available at 10.1186/s12913-023-09466-9.

## Background

Bridging the gap between researchers and the researched and linking communities to valuable resources is key to community empowerment. Community Engagement (CE) or Empowerment is increasingly becoming critical across contexts and an integral part of most research endeavours [1]. Broadly, World Health Organisation (WHO) defines CE as a process that allows people to be actively and genuinely involved in defining matters that concern them, to make decisions about factors that affect their lives, in addition to formulating and implementing policies, planning, developing and delivering services, and to take measures to improve desired change [2]. Similarly, CE has been described as a process of working with and through groups of people affected by geographical proximity, special interests, or similar situations to address issues affecting their well-being or to identify priorities, resources, needs and solutions in the community in such a way that representative participation, good governance, accountability, and peaceful change are encouraged [3]. CE, particularly in research settings, is a dynamic research strategy that evaluates what role communities impacted by the issue under investigation should have in the research process itself. Therefore, CE in research may be viewed as a continuum, since it fits within a range of study designs and should not be viewed as a singular method. It is also a participatory research technique, in which communities participate equally in all research decision-making, at one end of the spectrum. However, even when a completely participatory design is not suitable or practical, there are several additional considerations for communities to be involved or engaged in research (e.g., in some basic science or biomedical research). The criteria for including CE in research can be employed with any research methodological approaches, including qualitative methods, quantitative methods, and the analysis of secondary data. In the same manner, the concepts of CE in research can be applied to all areas of health research, such as clinical research, laboratory science, and epidemiological studies. Community engagement in research occurs on a continuum. This continuum includes initiatives that are totally academic led with little community engagement, as well as those that are wholly created and executed by community members and/or groups. The most truly community-integrated and involved research on this continuum is classified as participatory research, community-based participatory research (CBPR), or participatory action research (PAR). In this paper, community-based health research approaches are described as those that strive to integrate scientifically sound principles with community-centred theories of change and efforts by communities to address pressing community health concerns.

There have been many studies on community engagement and empowerment in dealing with health issues [4-8]; however, these studies have not been rigorous. A study by Chen, Mullins, Novak, and Thomas [9] shows the importance of patient activation and empowerment as a cyclical process through a patient’s accumulation of knowledge, confidence, and self-determination for their health. These authors suggested an empowerment framework (from individual to a health care professional to community and health care delivery system-level), intended to inform the development of culturally informed personalized patient activation and empowerment (P-PAE) interventions to improve population health and reduce racial and ethnic disparities [9]. Another study found peer navigators useful for addressing health problems through community-based participatory research (CBPR) in ensuring that communities are empowered on health issues [10]. Core concepts and principles of CBPR were also found to be effective in the management of health issues at the community level [11]. CBPR is the oldest and best-known approach under the umbrella term of community-engaged research (CEnR) used to represent a variety of activities and methodologies (e.g., stakeholder engagement, patient engagement, public involvement, and participatory action research). Nevertheless, all CEnR approaches draw from CBPR's emphasis on including community members as equal partners in many aspects of the research process, from the identification and selection of priority topics and research questions to the development of data collection materials and analytical strategies to the drafting and dissemination of the publication of findings [5].

CE in research has been proven to increase the impact of health studies on communities on several occasions. This way, research demonstrates greater sensitivity to the perception, needs and unique circumstances of researched communities. Many studies have pointed out the need to better apply research, disseminate research results more effectively, and synthesize research into evidence-based guidelines and "best practices" for more immediate use by practitioners and community members [12-15]. In line with these observations, CE has been considered crucial and pivotal, as involving communities in research settings can make findings and recommended interventions more relevant to local needs, informed by local knowledge and priorities, and thus more effective [16, 17]. An empowered community is one that can identify its own needs and have the capability to raise questions and issues with others; has opportunities to make choices or influence decisions being made by others on its behalf; as well as can stimulate and monitor actions in pursuit of the decisions that were made or influenced by the community. Existing literature underlines the need for improved practice, coordination, integration, and measurement of community engagement [18]. These are essential components of community systems strengthening, with multifaceted implications and demonstration of intervention effectiveness, responsiveness, and accountability across sectors.

From the definitions of CE, it is evident that the concept of “community” is central. However, there are many types of communities [19]. Communities are typically described as geographical, as in settlements or parts of town, but they are not always territorial. They can also include groups of people who share common practices or goals (such as investment or advocacy groups) and can exist in a variety of settings (e.g., virtual or physical spaces) [17]. In this sense, a community has been defined as a social unit that is locally relevant just above the level of the household (i.e., neighbourhood, town, parish). A community may also include non-geographically focused social networks of interaction, exchange, and interdependence. Such networks could contribute to the transfer of health, education, social, information, economic, cultural, and political resources [18]. Depending on the socio-economic and political circumstances at the time, CE can be transformative, supporting marginalized groups in empowering and emancipating themselves. Community mobilization that ignores suitable CE tactics and their intended consequences, on the other hand, might detract from community participation's goals, aggravate exclusionary practices, and further entrench disparity.

While CE remains an important aspect of research it is one of the most less understood and less funded aspects of the research process [19]. When grant writers seek funding for research the critical steps which require community engagement have already been surpassed. The funder requires to see a complete proposal with the problem already identified, funders also anticipate that the researchers have done the necessary consultations with the researched communities to identify problems together. This initial step is important; however, researchers intentionally skip this step as it requires more resources such as time and funding. The CE process is unique in the sense that it happens prior to the research, then it comes in during the research process and continues after the research has ended. Most funding can only support CE during the research process because the funding will be available by then, otherwise prior to the release of funds and post the reporting stage funding is not available. This unavailability of funding is one of the key barriers which limit the implementation of CE by many researchers. Another limitation is that most researchers are not trained or oriented systematically to the process of CE. Quite often researchers miss the key step because they are not aware of it, or they simply do not have the expertise to go about it. Therefore, training in community engagement is just as important as training in any other field and it has the potential to shape the future conceptualisation, implementation and impact of CE research [20]. This is further coupled by the fact that some funders are reluctant to release funds to support the development of skills and awareness around CE. The bulk of researchers only focus on dissemination skipping all so important process of CE.

Studies on approaches to community engagement and empowerment have mostly focused on aspects of community consultation (decision-making processes) but less on the other crucial, equally important elements: assessing how the community’s capacity is developed; and, ensuring that implementation follows and is in accord with the consultation and decision-making process. The growth in interest and utilization of CE in research, therefore, raises the more compelling question of the actual outcomes of community participation and involvement. What kinds of evidence are there to support the CE's substantive outcomes? What have been social-ecological impacts on local communities where CE has been implemented? These general questions, however, are linked to a more significant question: what are the various CE processes, strategies and approaches that have been adopted in research settings? The purpose of this systematic literature review was to review the literature on the processes, strategies and approaches of community engagement and empowerment that have been adopted by researchers in low-income countries to determine the best practice for community engagement and empowerment. It is noticeable that most CE definitions refers to CE as a process/strategy/approach, and these three concepts are considered to be synonymous; these focus on single specific hypothesised processes drawn from the community engagement framework to identify how one phenomenon influences another. Hence, these concepts were used in this systematic literature review interchangeably as they were also part of the search terms. In line with these pertinent questions, this systematic literature review sought to understand the nature of CE processes in health research environments in Lower Middle-Income Countries (LMICs). LMICs refer to those countries defined by the World Bank based on the countries’ Gross National Income as having “low-income economies,” “lower middle-income economies” or “upper middle-income economies” as may be amended from time to time [21].

## Methods

This systematic literature review followed the Preferred Reporting Items for Systematic Reviews and Meta-Analyses (PRISMA) guidelines. Since this was primarily a qualitative systematic review, no specific comparator interventions or demographics were sought, and a wide range of study approaches, whether experimental, descriptive, or exploratory/explanatory, were considered. We predominantly considered qualitative, quantitative, and mixed-methods studies. All of the articles ultimately included in this systematic literature review were based on empirical research. In accordance with our objectives and problem statement, we employed thematic data analysis which is qualitative in its nature. The nature, scale, and intended effects of CE processes, strategies and approaches in research environments were among the domains reviewed. The systematic literature review did not explore all the models that are available in the literature, we limited our scope to certain criteria to achieve the systematic literature review purpose. Due to the limited amount of time that was available to conduct the review and the limited scope of the review, we avoided exploring other existing models. An extensive search of the models was conducted, but we were restricted in what we could access. We only included records that were publicly available. Some of the records in the databases where we were searching for our records were not available for free, limiting our record selection.

### Information sources

A literature search was carried out in three electronic databases: PubMed, Web of Science and Google Scholar in December 2021. Each database was searched from January 2011 to December 2021 for articles containing concepts related to community, engagement, research settings and LMICs as shown in Table 1. We searched for the latest findings and latest evidence in literature within the past 10 years to track trends of the studied phenomenon. Studies reviewing the same topic have been conducted and we needed latest findings on this topic area. Beyond that, the evidence would become obsolete. Recent studies are, therefore, highly relevant to the research question. Apart from searching databases, we have also looked for references that were included in the citation we have found.

**Table 1:** Concepts and associated terms used as search terms in the literature search

Concept	Search Terms
Community Engagement	"Community Networks"[Mesh] OR "Community "[text word] OR “Communities” [text word] OR "Community Research Planning"[Mesh] OR "Community-Institutional Relations"[Mesh]
Process	Process OR Strategies OR Approaches
Community Engagement Outcomes	“Research Partnerships”, Accountability, “Project Ownership”, “Community Empowerment”, Sustainability
Research Settings	"Health Services Research" [Mesh] OR "Community-Based Participatory Research" [Mesh] OR "Operations Research" [Mesh] OR “Qualitative Research” [Mesh] OR "Evaluation Studies as Topic" [Mesh] OR "Evaluation Studies" [Publication Type] OR "Health Care Evaluation Mechanisms" [Mesh] OR "Program Evaluation" [Mesh]
LMICs	"Lower-middle-income economies"[tiab] OR “low income economies”[tiab] OR "Developing countries"[mh] OR "developing countries"[tiab] OR "developing country"[tiab] OR "under-developed countries"[tiab] OR "under-developed country"[tiab] OR "third-world countries"[tiab] OR "third-world country"[tiab] OR "developing nations"[tiab] OR "developing nation"[tiab] OR "underdeveloped nations"[tiab] OR "third-world nations"[tiab] OR "third-world nation"[tiab] OR "less-developed countries"[tiab] OR "lessdeveloped country"[tiab] OR "less-developed nations"[tiab] OR low and middle income countries[tiab] OR lmic[tiab] OR low income country[tiab] OR low income countries[tiab] OR lower income countries[tiab] OR middle income country[tiab] OR middle income countries[tiab] OR lower middle income country[tiab] OR lower middle income countries[tiab] OR “Afghanistan” … Zimbabwe[tiab]

### Data collection process and data items

In Google Forms, a data extraction form was constructed to help extract information from each article on essential components of community engagement processes, strategies, and approaches in research settings, as listed under the research objectives. The study quality was assessed using the Critical Appraisal Skills Program (CASP) and elements of rigour in doing research [22]. Critical appraisal skills program is a central process to evidence-based practice and is used in reviewing scientific papers to determine good research from bad research. Critical appraisal has two main roles which are, first to eliminate studies of low quality which have results that may compromise the validity of the research; secondly, to identify the strengths and limitations of included studies [23]. The manner in which critical appraisal is done is important in determining the quality of a systematic review, CASP is usually done at the stage of full text assessment. For qualitative papers its aim is to call out the rigor of research papers and determine levels of transferability, while for quantitative papers its purpose is to reduce the risk of bias or misleading readers [23]. From CASP, four broad categories were derived for study quality assessment, these were: sampling, data collection, analysis, and trustworthiness—reliability (or dependability) and validity (or credibility) of the research. Reliability refers to the extent to which results are repeatable under different conditions, validity refers to the extent to which the measures or mirrors the concept being researched on [23]. Mthembu and Mogaka from the review team independently piloted the form by abstracting two sample articles. The form was revised and further improved after a collective review and discussion. We refined the google form based on the objectives, inclusion and exclusion criteria. Once we had the inclusion and exclusion criteria, we tested it by searching the databases. Then we looked at the results whether they were of satisfaction, if not, we refined the form again. This was an iterative process that involved going back and forth trying to find what works best. Therefore, the form was changed to adjust it to what was going to produce the results in terms of outcomes of the review. The reviewers came to an agreement on how to abstract the remaining articles. The remaining articles were abstracted, and the researchers had regular online sessions to discuss new findings, issues encountered throughout the abstraction process, and a consensus approach to resolving them.

### Analysis

**Fig. 1:**
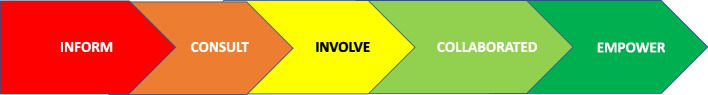
Community engagement vancouver coastal health framework (source: www.vch.ca/ce)

Findings were synthesized using a thematic approach, commonly used in systematic reviews to summarize qualitative and quantitative studies [24]. This systematic literature review applied the Vancouver Coastal Health Community Engagement Framework (www.vch.ca/ce) which involves five CE distinct stages (see Fig. 1). The stages include informing the community, consulting the community, involving the community, collaborating with the community and empowering the community. In this systematic literature review, these stages were interpreted as whether communities were (a) informed about the problems and solutions proposed in the research; (b) consulted in identifying and defining the problems and interventions designed to address those problems; (c) involved through participating in the implementation of research projects; (d) collaborated with in managing research project resources e.g. monitoring and evaluation; and/or (e) Empowered in taking ownership of the research process. Abstracted findings were synthesized into detailed outputs after articles were revisited multiple times. Following a process of constant comparison, two reviewers (Mthembu and Mogaka) reviewed and amended them in consultation with the supervisor (Chimbari). The design and analysis of the systematic literature review were also evaluated and reported on for quality using CASP.

## Results

### Article selection

**Fig. 2:**
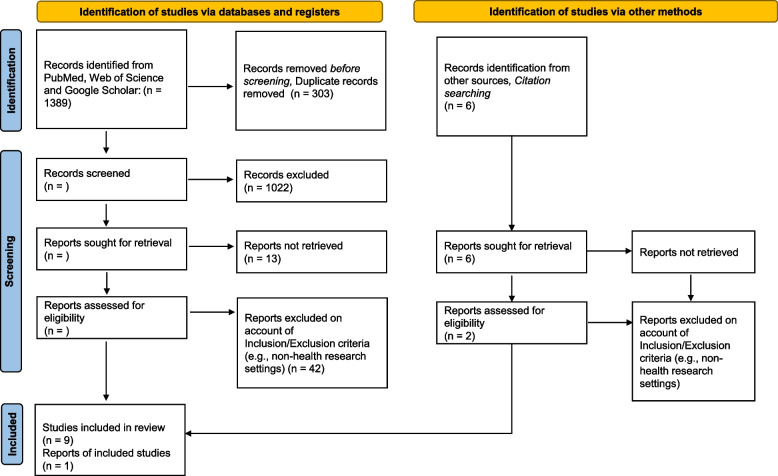
PRISMA flow diagram for the systematic literature review

As shown in the PRISMA flow diagram in Fig. 2, the result of our search yielded 1,389 articles, which after 303 duplicates were removed, left a total number of 1,086 articles. 1022 were excluded at title/abstract stage. This meant that 64 articles were sought for retrieval. However, of these, 51 were assessed for eligibility because 13 could not be retrieved. Some articles were simple not available as the authors did not post them online or posted a citation not the actual paper and we did not have access to authors. At this stage, this had a minimal impact to understand the studied phenomenon, however we did miss the chance to find papers which could possibly qualify for the main review. Of the 51 records assessed for eligibility 42 were further excluded as a result of low levels of CE throughout the study cycle, non-health research settings. This left 9 articles that were finally admitted for data extraction. A further six articles were identified through the references section of the admitted articles. However, four of these could not be retrieved and one was excluded after applying the inclusion/exclusion criteria. This left one article admitted for data extraction. In total, therefore, ten articles were finally examined, and data extracted on CE processes. Research settings and CE outcomes of intent.

### Article characteristics

**Fig. 3:**
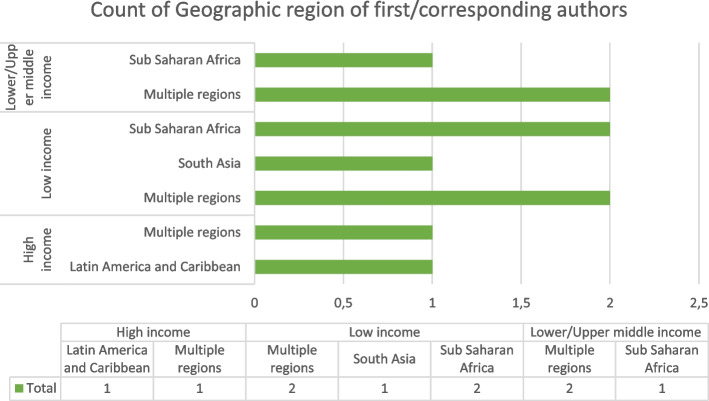
Study characteristics of geographic regions of first/corresponding authors and study location

The geographic location where the CE processes/strategies/approaches were studied/implemented were assessed. We also assessed where and who publishes health research in LMIC that involves community engagement. When two different affiliations were mentioned, only the first affiliation mentioned was categorized. Locations were divided into 3: Low-Income, Lower/Upper Middle-Income, and High-Income countries. Regions, where CE strategies were implemented/studied according to surveyed literature, were divided into 4: Sub-Saharan Africa, Latin America and the Caribbean, South Asia or simply Multiple regions if the focus of the reviewed paper was global in nature. The results are illustrated in Fig. 3.

Despite the emphasis on LMICs, some of the first/corresponding authors were from high-income nations. Among those articles whose first/corresponding author was based in an LMIC region, [70%] which is 7/10 focused on either multiple regions or Sub-Saharan Africa. Moreover, it was noted that half of these articles were authored by those in Low-Income countries.

#### Health research domains

**Fig. 4:**
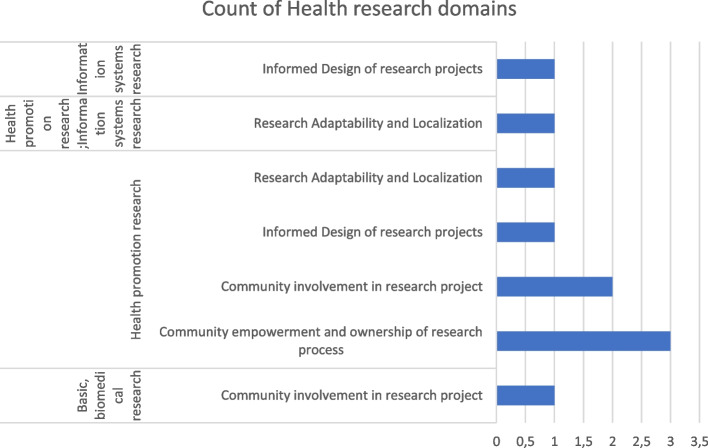
Study characteristics of geographic regions and health research domains

The assessment was based on which health research domains had strategies that involved community engagement and identified only three major research fields: Basic Biomedical research, Health Promotion research and In-formation Systems research (Fig. 4). As shown in Fig. 4 and Table 2, engaging communities in health promotion was most prevalent in Sub-Saharan Africa, with over 50% of the research that took place in Sub-Saharan Africa and/or whose focus was also on multiple regions mainly featuring this type of research. However, Sub-Saharan Africa also featured Basic Biomedical and Information Systems research. The majority of the health-related promotion articles in this review included community participation in research activities mainly in community involvement in the research process and empowerment but were less likely to have communities defining the problem that needed to be addressed, defining the intervention to be recommended, managing resources for it or monitoring/evaluating the research project. Information Systems was the health research domain with the fewest articles [10%] which is 1/10 and the least participatory with regard to community empowerment and ownership of research processes, among other CE outcomes of interest at [20%] which is 2/10.

**Table 2:** Summary of systematic literature review findings

First/Corresponding Author (Surname and Name) and Year of Publication	Geographic region of First/Corresponding authors	Geographic region of CE process/strategy/approach	Health research domains	Type of health condition researched	Process or stage of CE described in the article?	CE approach/method used
Chaka Chirozva, 2016 [25]	Low income	Sub-Saharan Africa	Basic, biomedical research	Environment	1 Consultation in identifying or defining problems & interventions 2. Involvement in a research project	Focus group discussions
Chiao-Wen Lan, 2017 [26]	Low income	Multiple regions	Health promotion research	Reproductive and child health (Maternal, Under-five/Newborn, Family planning)	1. Consultation in identifying or defining problems & interventions 2. Empowerment and ownership of research process	
Irene Jao, 2015 [27]	Low income	Sub-Saharan Africa	Health promotion research	Other broader health determinants, Broader health issues, primary care	1. Informing communities about problems & solutions proposed 2. Consultation in identifying or defining problems & interventions 3. Collaboration with community in monitoring, evaluating research projects 4. Empowerment and ownership of research process	Community research project oversight groups; Community awareness campaigns
Greer Haintz, 2019 [28]	Lower/Upper middle income	Multiple regions	Health promotion research	HIV and Other sexual and reproductive health	1. Informing communities about problems & solutions proposed 2. Consultation in identifying or defining problems & interventions 3. Empowerment and ownership of research process	Education, information-education-communication (IEC) campaigns, behavior change, peer-led, participatory, empowerment-based, communication campaigns, social and behavioral communication campaigns, and mass-media campaigns
Sheri A. Lippman, 2017 [29]	Lower/Upper middle income	Multiple regions	Health promotion research	HIV and Other sexual and reproductive health	1. Informing communities about problems & solutions proposed 2. Involvement in research project 3. Empowerment and ownership of research process	Partnership building; groups/networks engagement (community action teams); workshops, print material dissemination, Short films (Digital stories); Door to door outreach screening
Jose A. Arriola-Vigo, 2019 [30]	High income	Latin America and Caribbean	Health promotion research	Non-communicable disease other conditions	1. Consultation in identifying or defining problems & interventions 2. Collaboration with community in monitoring, evaluating research projects 3. Empowerment and ownership of research process	Employing community mental health workers; home visits; psychosocial clubs; mental health workshops and campaigns; peer support groups
Irene Jao, 2015 [31]	Lower/Upper middle income	Sub-Saharan Africa	Health promotion research, Information systems research	Broader health issues, primary care	1. Consultation in identifying or defining problems & interventions 2. Collaboration with community in monitoring, evaluating research projects	
Osama Ahmed Hassan, 2017 [32]	Low income	Multiple regions	Health promotion research	TB and Other infectious diseases	1. Informing communities about problems & solutions proposed 2. Consultation in identifying or defining problems & interventions 3. Empowerment and ownership of research process	Community consultation through Community Surveys; Face-to-face Interviews with household heads
Kimberly Baltzell, 2019 [33]	High income	Multiple regions	Health promotion research	Malaria	1. Informing communities about problems & solutions proposed 2. Involvement in research project 3. Empowerment and ownership of research process	Thought leaders (defined as those with expertise or leadership positions in sectors included in the study); Focus group discussions (FGDs); Community meetings; Drama and music performances, art shows, and school-based activities; Door-to-door engagement; Community motivation
Abhay Gaidhan, 2020 [34]	Low income	South Asia	Information systems research	Environment	1. Consultation in identifying or defining problems & interventions	Photographs and stories

#### Types of health conditions

The assessment was based on which types of health conditions were most likely to be addressed through research that involved community engagement. Community participation was most frequently observed in research concerned with infectious diseases, including Vector-Borne Diseases (VBDs) e.g., Malaria, human immunodeficiency virus (HIV) and tuberculosis (TB) [40%], which is 4/10, followed by articles presenting studies on the environment [20%] which is, 2/10 as well as those pertaining to other broader health determinants, broader health issues and primary care [20%], which is 2/10. The least health conditions that were investigated and at least involved some degree of CE were found in studies concerned with reproductive and child health (Maternal, Under Five/Newborn, Family planning) [10%] as well as those concerned with non-communicable disease and other similar conditions [10%].

### Process or stage of CE described in the article

The findings on type of CE strategy used were classified and presented following the Vancouver Coastal College CE framework. The classifications helped us to determine whether each study was able to articulate the 5 stages of the community engagement cycle. Here we state each part of the cycle and the articles that applied each stage/process in their study: (a) community informed about the problems and solutions proposed in the research [28, 29, 31-33]; (b) community consulted in identifying and defining the problems and interventions designed to address those problems [25-28, 30-32, 34]; (c) community involved through participating in the implementation of research projects [25, 29, 33] (d) community collaborated with in managing research project resources e.g. monitoring and evaluation [27, 30, 31]; (e) community empowered in taking ownership of the research process [27-30, 32, 33].

### Type of CE method or approach

The broad range of methods and approaches for CE were assessed and outlined as mentioned/discussed in surveyed literature. These techniques varied based on whether communities were informed, consulted, involved, collaborated with or empowered. Table 2 summarizes the major techniques for CE included. 4 main categories were identified and are explained below:

1. CE through Artwork and Creativity
  - Photography: Photography was seen as an essential and successful technique for integrating people into health-related research activities [34]. Images integrate living experiences with scientific knowledge, allowing individuals to relate to health messaging. Photographs can also be used to represent health-related demands and opinions without the use of complicated language or scientific understanding.
  - Short films and digital story screening: these were also seen as effective means of engaging the community [13].
  - Communities were also engaged through festivities that involved songs, poems and similar artworks [33]. These artworks described the area of the community, changes members would like to see, and their ideal environment.

2) CE through Workshops and Focus Groups

Workshops [13, 35] and focus groups [6, 33] were seen as avenues that allow people to discuss their ideas in an open and relaxed atmosphere. There were a variety of workshop formats described in the surveyed literature. Some workshops were meant for the exchange of information; others to discuss the strengths, weaknesses, opportunities and threats of an idea or project, while others were for obtaining ideas and innovative thinking for a way forward for a project. Focus groups by contrast are designed to specifically concentrate on a single issue. Both workshops and FGDs were avenues for education, information-education-communication (IEC) campaigns, behaviour change, peer-led, participatory, empowerment-based, communication campaigns, social and behavioural communication campaigns, and mass-media campaigns [28].

3) CE through forums

A forum was seen as a regular meeting of people who represent a group of organizations and were issue or area based. Those involved typically comprised members of civic, political, professional, economic or social groups from a local area. A number of articles [13, 27, 35] referred to community leader forums as key influencer forums, and that research programs are likely to fail if local leadership does not support the program’s goals.

4) CE through community surveys

Questionnaire surveys were seen as means of identifying the needs and views of a large number of people in a standard format [32].

### Quality of study design and analysis

**Fig. 5:**
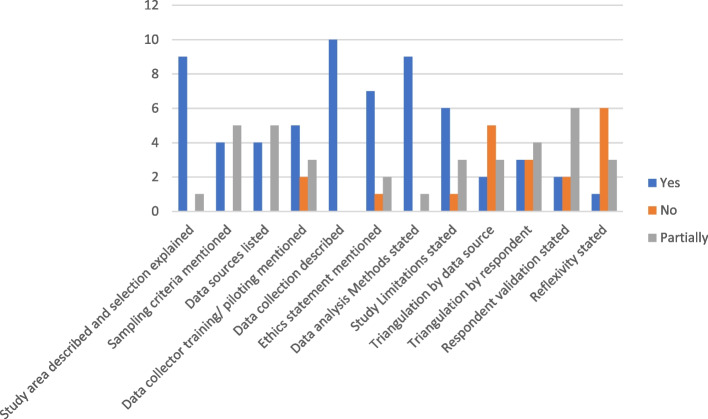
Systematic literature review Quality assessment results

Several components of the study quality-examined were clearly unsatisfactory when it came to the interpretation of responses to the quality of study designs and data analysis. Independent of the data collection description, no article fulfilled every requirement for a research article. At least 60% of studies provided basic aspects related to describing study area and selection, data sources and collection, and triangulation across data sources. Despite this, 30 percent of articles did not have declaration of ethical approval from review boards, while 60% of the papers acknowledged limitations (Fig. 5). The quality of studies varied depending on their study designs. No study could possibly list all of the characteristics of good quality design that we discussed, some studies did not adequately list their sample criteria [25, 27, 31, 32, 34], study participation rates [28-31, 33, 34], ethics statements [6, 26, 31], only two studies adequately mentioned respondent validation [29, 34], and only one study mentioned researcher reflexivity [28]. (See supplementary table 1 in the supplementary material showing how each study rated in the study quality assessment).

## Discussion

A distinguishing mark of the CE research literature in health research settings is that more needs to be done at a variety of levels. For community engagement in LMICs as previously de-fined, we have found very few published studies of empirically collected CE processes data [26, 27, 29], and in fact, few published CE processes studies of any kind, in professional journals. Some related evidence comes from studies of non-research settings of community engagement projects and initiatives [28, 34].

Although our analysis included studies that are simply qualitative, quantitative, or mixed methods, the majority of studies were exploratory in origin, with only a few using probability or experimental designs to evaluate or describe health research in relation to community engagement. Some reviews on community participation have also noted the scarcity of experimental designs that examine the effectiveness of community participation [36, 37], others have called to attention the lack of process evaluations [7] and qualitative research [38] to investigate more thoroughly how community participation contributed to the health outcomes linked to it. The challenge in noticing such an effect has previously been ascribed to insufficient distinction between CE as a continuous social process, not as a once-off or now-and-again community intervention, a process that requires alternative evaluation models [19, 39]. Given the variable study quality of many of the publications in this systematic literature review, as well as the challenge of assessing community engagement, due to its multi-faced, context-specific, and contentious nature, more high-quality research is needed to better understand the CE processes or strategies.

### CE approaches

Several CE approaches were presented in the surveyed literature. However, a deeper discussion on the appropriate contexts where each can be used was lacking across all articles. For instance, the utility of Arts and Creative approaches for CE are very appropriate where local people are involved in expressing their views and generating ideas in a participative approach. They are a useful technique for engaging with people of all ages through education or school programs, local community forums and resident or interest groups. However, they are specifically beneficial at the beginning of a community research project in the planning process to generate interest and raise awareness of the projects. Moreover, this technique often depends on the availability of a large space to exhibit or display results. It may also be difficult to interpret participants’ ideas in this mode of CE. It is significant to understand cultural dynamics in the community engagement discourse as culture provides a sense of belonging in the community, shapes identities, and affects how people interact with one another, define power, and generate meaning [40]. Culture also affects perceptions of collaboration, trust, and negotiation. As a result, culture influences how communities are engaged, and cultural knowledge is necessary for successful community engagement [41]. Therefore, researchers and practitioners need to understand the cultural dynamics of specific groups and institutions in order to build relationships, identify ways to effectively collaborate, and build respect and trust. Workshops and focus group discussions are useful approaches for encouraging discussion among those who may feel less confident in larger group meetings, with the benefit of targeting participants or certain interest groups. However, even though this has the advantage of identifying and inviting those often excluded from a wider engagement exercise, with such small groups, it is often difficult to be sure all stakeholders or interests are represented. Workshops can also be dominated by articulate and confident individuals if not carefully facilitated, unless experienced facilitators are present.

Many of the assessed articles presented creative and socially based approaches to showcase CE. However, utilizing multiple channels of communication is important. Examples of other techniques and channels for CE include the use of soccer clubs, films, community drama, television, songs, Facebook, social events such as weddings, music festivals, and community fairs with prizes. Children were repeatedly mentioned as important change agents and it was recommended that youth groups be mobilized to assist with messaging. This is one way of ensuring that a part of the community actually participates in the role of the research. Mobilizing groups of young people to become citizen scientist or assistant researchers can significantly improve the involvement of young people, it also brings in the element of collaborations especially when young people bring in their ideas and expertise in solving some of the problems as they work with the researchers. CE borders mainly informing, consulting, involving, collaborating and empowering, hence it is important to have an approach that can accommodate all or most of these stages. Drama and creative art facilitate the process of informing, involving but lacks consultation, collaboration and empowerment. Workshops and FGDs bring on the aspect consultation but still lack in collaboration and empowerment unless the workshops take the form of trainings. Peer educators, community forums (such as community advisory boards) and other forms of collaboration including working with community health workers and community research assistants enable the implementation of all the five stages of the CE process framed by the Vancouver Coastal College CE framework (informing, consulting, involving, collaborating and empowering).

### Implications of CE in lower-and middle-income countries

The review shows that the understanding of implementing principles of CE in researching health-related issues is still in its developmental phase, which poses threats to recognising communities from LMICs as knowledge experts. This review demonstrated that there is limited literature on empirical research published on CE processes in relation to health issues in LMICs. Furthermore, the dearth of empirical research in this study subject calls for more measures to conduct empirical studies to close the gaps between a theoretical perspective and its application in the real world to determine the relevance of CE strategies and approaches to address social issues. Some scholars raised great concern by describing CE as less participative and lacking theoretical components that are responsive to the needs of the researched communities in LMICs [6, 13, 27, 35, 42]. However, this is debatable because LMICs have different characteristics that researchers need to consider. Sometimes researchers in LMICs dominate the research processes, without full or direct participation from researched communities. Another realisation is that some of the CE approaches are not applicable and responsive to the needs of people in LMICs when dealing with health issues in a research context. This suggests the reorganization or reconsideration of the assets that exist not only as regards the content or principles and the methods, but a rethinking of various phases and processes of CE [43, 44]. Moreover, these gaps pose a threat to the need to envision CE as a participatory approach in which all components, including understanding the concepts, theories and processes are considered when conducting engaged research on health-related issues in LMIC.

This review highlights how crucial it is for researchers conducting community engagement health studies in LMICs to take into consideration the level and the nature of community participation. There is a need for high-quality study designs to better understand the CE processes and approaches in achieving successful community engagement. A key area that emerged from the systematic literature review is that most CE techniques are creative in nature and socially based but the appropriateness, feasibility and value of these techniques are not considered when dealing with health issues in LMICs. Therefore, participatory approaches and strategies, such as community based participatory research (CBPR) being the most established and well-known in the health field should be recognised and adopted in CE. This could provide more in-depth insights into community engagement in health interventions as well as the connections between variables such as power dynamics and their impact on community participation outcomes. Previous studies that adopted CBPR in dealing with health initiatives found CBPR to be one of the most successful strategies for improving community participation in rural settings [5, 45, 46]. These findings suggest that researchers should identify the best CE processes, strategies and approaches that are most appropriate to community settings when dealing with health interventions in LMICs.

### Limitations

We faced limitations during the literature search where some studies that qualified for abstract screening were not available or accessible for free (were available on purchase) and we did not have funding to buy the articles, our institutional library also did not have access to these papers. Because of the unavailability of funds to pay to access these articles, we could not access them or use them in the next stages of the review. The institutional access which our university offered was not covering these journals. Other reasons for not accessing papers included the fact that some papers were listed as citations, but no full papers were available to view them, this could be a problem with wrong referencing or broken links to the source of the articles. In total we failed to access at least 13 papers which impacted the possible outcomes of our review. Another limitation of the review is that grey literature was not looked for. We only focused on peer reviewed and published articles. These limitations slightly reduced the scope of papers included the review and they may have affected the analysis and outcomes in general. Another limitation of the systematic literature review was the use of only the first institutional affiliation, given that two separate affiliations for the first and/or corresponding author were mentioned. This might have resulted in a measurement inaccuracy in terms of determining which economic region to adopt in classifying the study. However, the first author of a publication is typically the student/researcher who conducted the research, while the corresponding author is typically the senior author who gives intellectual input and prepares and approves the procedures to be used in the systematic literature review. We suggest the attribution as provided in this work is fair because it is based on the first or corresponding author of the selected publication.

## Conclusions

Even though reviewed works were relatively rigorous and appeared balanced, the findings on CE approaches and strategies were inconclusive. While desired change does sometimes occur, overall, the documented research evidence for positive coalition or partnership outcomes is weak. The reviewed publications contain some great examples of CE approaches and strategies, they also contain key cautionary components. Despite the importance of community participation and its history, there is still a lack of general understanding of the concepts, motivations, and social processes that drive it. Many publications are undertheorized and uncritical, with few citations to definitions or frameworks. While this may not appear to be relevant to the social transformation or utilitarian goals that motivate community engagement initiatives, it can help to explain the assumptions that underpin the type of community participation project that is supported, as well as clarify expectations about the scope of change that is expected, and the techniques required to achieve it on multiple levels.

## Supplementary Information

**Additional file 1: Supplementary Table 1.** Study quality assessment and appraisal table.

## Data Availability

The datasets used and/or analysed during the current systematic literature review available from the corresponding author on reasonable request.

## Abbreviations

CASP: Critical Appraisal Skills Program
CBPR: Community-Based Participatory Research
CE: Community Engagement
CEnR: Community-Engaged Research
IEC: Information-Education-Communication
LMICs: Low- and Middle-Income Countries
PAR: Participatory Action Research
P-PAE: Patient Activation and Empowerment
PRISMA: Preferred Reporting Items for Systematic Reviews and Meta-Analyses
VBDs: Vector-Borne Diseases
WHO: World Health Organisation

## References

[1] Ahmed SM, Palermo AGS (2010). Community engagement in research: frameworks for education and peer review. Am J Public Health.

[2] Organization WH (1997). Twenty steps for developing a healthy cities project.

[3] McCloskey DJ, Akintobi TH, Bonham A, Cook J, Coyne-Beasley T. Principles of Community Engagement (Second Edition). COMMUNITY Engagem. :197.

[4] Irish E, Burke K, Geyer E, Allard I (2022). Patient Empowerment: A Partnership for Community Engagement in Three Phases. J Consum Health Internet.

[5] Luger TM, Hamilton AB, True G (2020). Measuring community-engaged research contexts, processes, and outcomes: a mapping review. Milbank Q.

[6] Chirozva C (2016). Community engagement in the governance of Transfrontier Conservation Areas: An analysis of the implementation of Sengwe Tshipise Wilderness Corridor, Zimbabwe [Doctoral Thesis].

[7] Farnsworth SK, Böse K, Fajobi O, Souza PP, Peniston A, Davidson LL (2014). Community engagement to enhance child survival and early development in low- and middle-income countries: an evidence review. J Health Commun.

[8] Estacio EV (2013). Health literacy and community empowerment: it is more than just reading, writing and counting. J Health Psychol.

[9] Chen J, Mullins CD, Novak P, Thomas SB (2016). Personalized strategies to activate and empower patients in health care and reduce health disparities. Health Educ Behav Off Publ Soc Public Health Educ.

[10] Corrigan P, Pickett S, Kraus D, Burks R, Schmidt A (2015). Community-based participatory research examining the health care needs of African Americans who are homeless with mental illness. J Health Care Poor Underserved.

[11] R X, Jr S, Je H, Sg K. Promoting Community Health and Eliminating Health Disparities Through Community-Based Participatory Research. Phys Ther. 2016 ;96(3). Available from: https://pubmed.ncbi.nlm.nih.gov/26251479/. [Cited 2022 Dec 15]. 10.2522/ptj.20140529 26251479

[12] MacQueen KM, McLellan E, Metzger DS, Kegeles S, Strauss RP, Scotti R (2001). What is community? An evidence-based definition for participatory public health. Am J Public Health.

[13] Lippman SA, Pettifor A, Rebombo D, Julien A, Wagner RG, Kang Dufour MS (2017). Evaluation of the Tsima community mobilization intervention to improve engagement in HIV testing and care in South Africa: study protocol for a cluster randomized trial. Implement Sci IS.

[14] Matthews AK, Anderson EE, Willis M, Castillo A, Choure W (2018). A community engagement advisory board as a strategy to improve research engagement and build institutional capacity for community-engaged research. J Clin Transl Sci.

[15] Evans E, Funes M, Hong H, Reuter K, Ahmed S, Calhoun K (2018). Defining and measuring community engagement and community-engaged research: clinical and translational science institutional practices. Prog Community Health Partnersh Res Educ Action.

[16] Nutbeam D (2000). Health literacy as a public health goal: a challenge for contemporary health education and communication strategies into the 21st century. Health Promot Int.

[17] Patrick DL, Wickizer TM. Community and Health. In B. C. Amick, S. Levine, A.R. Tarlov & D. C. Walsh (Eds.), Society and Health. New York: Oxford University Press; 1995. pp. 46-92.

[18] Bedson J, Skrip LA, Pedi D, Abramowitz S, Carter S, Jalloh MF (2021). A review and agenda for integrated disease models including social and behavioural factors. Nat Hum Behav.

[19] George AS, Mehra V, Scott K, Sriram V (2015). Community participation in health systems research: a systematic review assessing the state of research, the nature of interventions involved and the features of engagement with communities. PLoS One.

[20] Dudley L, Gamble C, Allam A, Bell P, Buck D, Goodare H (2015). A little more conversation please? Qualitative study of researchers’ and patients’ interview accounts of training for patient and public involvement in clinical trials. Trials.

[21] Lencucha R, Neupane S (2022). The use, misuse and overuse of the ‘low-income and middle-income countries’ category. BMJ Glob Health.

[22] Long HA, French DP, Brooks JM (2020). Optimising the value of the critical appraisal skills programme (CASP) tool for quality appraisal in qualitative evidence synthesis. Res Methods Med Health Sci.

[23] Porritt K, Gomersall J, Lockwood C (2014). JBI’s systematic reviews: study selection and critical appraisal. AJN Am J Nurs.

[24] Lucas PJ, Baird J, Arai L, Law C, Roberts HM (2007). Worked examples of alternative methods for the synthesis of qualitative and quantitative research in systematic reviews. BMC Med Res Methodol.

[25] Chirozva C. Community engagement in the governance of Transfrontier Conservation Areas: An analysis of the implementation of Sengwe Tshipise Wilderness Corridor, Zimbabwe. [Doctoral Thesis, Charles Sturt University]. Austrialia Charles Sturt University. 2016;1-326.

[26] Lan CW, Tavrow P (2017). Composite measures of women’s empowerment and their association with maternal mortality in low-income countries. BMC Pregnancy Childbirth.

[27] Jao I, Kombe F, Mwalukore S, Bull S, Parker M, Kamuya D (2015). Involving research stakeholders in developing policy on sharing public health research data in Kenya: views on fair process for informed consent, access oversight, and community engagement. J Empir Res Hum Res Ethics.

[28] Lamaro Haintz G. "Theorising community engagement in sexual and reproductive health promotion in South Africa." PhD dissertation, Deakin University. 2019;1-322.

[29] Lippman SA, Neilands TB, MacPhail C, Peacock D, Maman S, Rebombo D (2017). Community mobilization for HIV testing uptake: results from a community randomized trial of a theory-based intervention in rural South Africa. J Acquir Immune Defic Syndr 1999.

[30] Arriola-Vigo JA, Stovall JG, Moon TD, Audet CM, Diez-Canseco F (2019). Perceptions of community involvement in the Peruvian mental health reform process among clinicians and policy-makers: a qualitative study. Int J Health Policy Manag.

[31] Jao I, Kombe F, Mwalukore S, Bull S, Parker M, Kamuya D (2015). Research stakeholders’ views on benefits and challenges for public health research data sharing in Kenya: the importance of trust and social relations. PLoS ONE.

[32] Hassan OA, Affognon H, Rocklöv J, Mburu P, Sang R, Ahlm C (2017). The One Health approach to identify knowledge, attitudes and practices that affect community involvement in the control of Rift Valley fever outbreaks. PLoS Negl Trop Dis.

[33] Baltzell K, Harvard K, Hanley M, Gosling R, Chen I (2019). What is community engagement and how can it drive malaria elimination? Case studies and stakeholder interviews. Malar J.

[34] Gaidhane A, Holding P, Shah M, Patil M, Telrandhe S, Jadhav N (2020). Photostory—A “Stepping Stone” approach to community engagement in early child development. Front Public Health.

[35] Arriola-Vigo JA, Stovall JG, Moon TD, Audet CM, Diez-Canseco F (2019). Perceptions of community involvement in the Peruvian mental health reform process among clinicians and policy-makers: a qualitative study. Int J Health Policy Manag.

[36] Preston R, Waugh H, Larkins S, Taylor J (2010). Community participation in rural primary health care: intervention or approach?. Aust J Prim Health.

[37] Atkinson JA, Vallely A, Fitzgerald L, Whittaker M, Tanner M (2011). The architecture and effect of participation: a systematic review of community participation for communicable disease control and elimination Implications for malaria elimination. Malar J.

[38] Marston C, Renedo A, McGowan CR, Portela A (2013). Effects of community participation on improving uptake of skilled care for maternal and newborn health: a systematic review. PLoS One.

[39] Marston C, Hinton R, Kean S, Baral S, Ahuja A, Costello A (2016). Community participation for transformative action on women’s, children’s and adolescents’ health. Bull World Health Organ.

[40] Chapter 1: Useful Concepts | Principles of Community Engagement | ATSDR. 2018. Available from: https://www.atsdr.cdc.gov/communityengagement/pce_useful.html. [Cited 2023 Apr 21].

[41] Chapter 2: Before Starting a Community Engagement Effort | Principles of Community Engagement | ATSDR. 2018. Available from: https://www.atsdr.cdc.gov/communityengagement/pce_principles_starting.html. [Cited 2022 Jul 26].

[42] Lan CW, Tavrow P (2017). Composite measures of women’s empowerment and their association with maternal mortality in low-income countries. BMC Pregnancy Childbirth.

[43] Adhikari B, Vincent R, Wong G, Duddy C, Richardson E, Lavery JV (2019). A realist review of community engagement with health research. Wellcome Open Res.

[44] Nimdet K, Chaiyakunapruk N, Vichansavakul K, Ngorsuraches S (2015). A systematic review of studies eliciting willingness-to-pay per quality-adjusted life year: does it justify CE threshold?. PLoS One.

[45] Salimi Y, Shahandeh K, Malekafzali H, Loori N, Kheiltash A, Jamshidi E (2012). Is Community-based Participatory Research (CBPR) Useful? a systematic review on papers in a decade. Int J Prev Med.

[46] HoonChuah FL, Srivastava A, Singh SR, Haldane V, HuatKoh GC, Seng CK (2018). Community participation in general health initiatives in high and upper-middle income countries: a systematic review exploring the nature of participation, use of theories, contextual drivers and power relations in community participation. Soc Sci Med.

