# Supplementary material for: Community engagement processes in low- and middle-income countries health research settings: a systematic review of the literature
Source: BMC Health Serv Res. 2023 May 8;23:457. doi: 10.1186/s12913-023-09466-9 (PMC10169489; doi:10.1186/s12913-023-09466-9)
Supplement: Supplementary file 1 — Additional file 1: Supplementary Table 1. Study quality assessment and appraisal table. [file 12913_2023_9466_MOESM1_ESM.pdf]

Supplementary Table 1. Study quality assessment and appraisal table.

**First Author and Year of Publication**	Chaka Chirozva, 2016	Chiao-Wen Lan, 2017	Irene Jao, 2015	Greer Haintz, 2019	Sheri A. Lippman, 2017	Jose A. Arriola-Vigo, 2019	Irene Jao, 2015	Osama Ahmed Hassan, 2017	Kimberly Baltzell, 2019	Abhay Gaidhane, 2020
Study area described and selection explained	1	1	1	1	1	1	1	1	0.5	1
Sampling criteria mentioned	0.5	1	0.5	1	1	1	0.5	0.5	1	0.5
Data sources listed	1	1	1	0.5	0.5	0.5	0.5	1	0.5	0.5
Data collector training/ piloting mentioned	1	0	0.5	0.5	1	1	0	1	1	0.5
Data collection described	1	1	1	1	1	1	1	1	1	1
Ethics statement mentioned	0.5	0	1	1	1	1	0.5	1	1	1
Data analysis Methods stated	1	1	1	1	1	0.5	1	1	1	1
Study Limitations stated	0.5	1	0.5	1	1	1	0.5	0	1	1
Triangulation by data source	1	0.5	0	1	0.5	0	0	0.5	0	0
Triangulation by respondent	0.5	1	1	0	0	0	0.5	1	0.5	0.5
Respondent validation stated	0	0.5	0.5	0.5	1	0.5	0.5	0.5	0	1
Reflexivity stated	0.5	0	0.5	1	0	0	0.5	0	0	0
**TOTAL SCORE**	**8.5**	**8**	**8.5**	**9.5**	**9**	**7.5**	**6.5**	**8.5**	**7.5**	**8**
